# PEACOCK: A Map-Based Multitype Infectious Disease Outbreak Information System

**DOI:** 10.1109/ACCESS.2019.2924189

**Published:** 2019-06-21

**Authors:** Beakcheol Jang, Miran Lee, Jong Wook Kim

**Affiliations:** Department of Computer ScienceSangmyung University 35005 Seoul03016South Korea

**Keywords:** Government agencies, infectious disease, infectious disease outbreak system, online news, search query

## Abstract

A map-based infectious disease outbreak information system, called PEACOCK, that provides three types of necessary infectious disease outbreak information is presented. The system first collects the infectious disease outbreak statistics from the government agencies and displays the number of infected people and infection indices on the map. Then, it crawls online news articles for each infectious disease and displays the number of mentions of each disease on the map. Users can also search for news articles regarding the disease. Finally, it retrieves the portal search query data and plots the graphs of the trends. It divides the risk into three levels (i.e., normal, caution, and danger) and visualizes them using different colors on the map. Users can access infectious disease outbreak information accurately and quickly using the system. As the system visualizes the information using both a map and various types of graphs, users can check the information at a glance. This system is in live at http://www.epidemic.co.kr/map.

## Graphical abstract

PEACOCK user interface of risk level information.

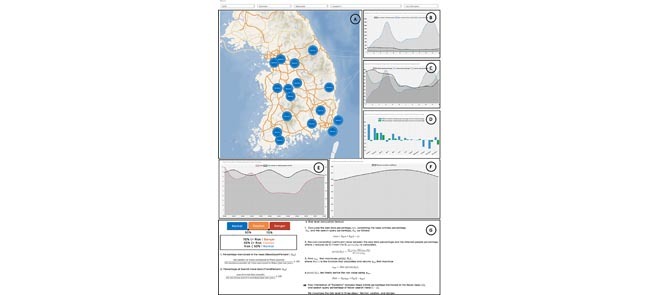

## I. Introduction

In 1918, the Spanish flu killed more than fifty million people [1]. Now, even after a century has passed, humanity is constantly threatened by infectious diseases. From severe acute respiratory syndrome (SARS) in 2003 to the H1N1 virus, Middle East respiratory syndrome (MERS), Ebola virus, and Zika virus, infectious diseases are still spreading. SARS caused 775 deaths, mainly in Asia [2], in 2009, and more than 18,000 deaths were recorded worldwide due to the swine flu epidemic [3]. According to the World Health Organization (WHO), by 2015, MERS had caused 1,800 deaths [4]. Ebola and Zika virus infections and fatalities have continued to increase. These infectious diseases cause not only human injury but also large-scale societal damage. From SARS in 2003 to the Zika virus in 2016, the world has suffered large economic and social damage owing to infectious diseases. Despite advances in medical technology and efforts towards the eradication of these diseases, there are still fewer than 30 infectious diseases with specific therapies, such as preventive vaccines. With the spread of mobile devices in recent years, there is a growing need for an infectious disease outbreak information system that monitors emerging diseases and provides information on the outbreak of infectious diseases.

Because of the importance of an infectious disease outbreak information system, many researchers and organizations have studied and developed such systems extensively [5]–[7]. Some systems provide infectious disease outbreak information based on statistical data collected by the Centers for Disease Control and Prevention (CDCs). Since the end of World War II, many countries have established their own CDCs for prevention and control of illnesses [8]. The information provided by those systems is true and accurate. However, CDCs rely on a centralized management system; hence, some lead time is necessary to collect and produce disease outbreak statistics. Consequently, a quick access to disease outbreak information becomes a challenge.

To generate and expedite disease outbreak information, several existing infectious disease outbreak information systems leverage web big data, such as online news media, portal search queries, and social network data [13]–[31], because an infectious disease that has repeatedly appeared in the news or been frequently searched for by users is likely to occur. These data are streamlined faster and information can be provided in real time. However, some systems proposed in previous studies [13]–[16], [18], [23], [27]–[31] only provide outbreak information for single or few infectious diseases, and users cannot check information for a wide range of infectious diseases. Other proposed systems [13], [16], [17], [19], [20], [28]–[30] only provide infectious disease outbreak information from a few kinds of data sources, so it is difficult to fetch the information quickly and accurately. Some systems [16], [30], [31] provide only text-centric information, which makes it difficult for users to understand the information at a glance. A few other systems [13]–[16], [18], [23], [27]–[31] are not in operation and cannot be accessed any more. The objective of this study is to implement an infectious disease outbreak information system that (1) generates and provides outbreak information for as many infectious diseases as possible; (2) exploits various data sources, such as CDC (i.e., KCDC), online news (i.e., Naver news), and web search queries (i.e., Naver search query); (3) uses various visualization tools, such as maps and figures rather than text only, and (4) remains in operation as long as possible. The contribution of this paper is as follows.

### A. Contributions of This Paper

We propose, develop, and operate an infectious disease outbreak information system, called PEACOCK, that is accurate, fast (real-time information from web big data, such as online news and portal search queries), user-friendly (map-based), and visual (combination and comparison of various types of infectious disease outbreak information). Our system provides the following three types of useful infectious disease outbreak information.

• PEACOCK provides the number of infected people per district on a map based on Korea Centers for Disease Control and Prevention (KCDC) data. It presents four types of infection indices and the fractions of infected people relative to population, district, and time. It also compares the number of infected people in the current month with that in the previous month and visualizes the status of the district in terms of the increase in infectious disease using a colored classification. In addition, it provides the number of infected people by district in a bar graph form.
• PEACOCK provides infectious disease information based on online news. Online news has been collected since August 2017. It provides the top-five most frequently mentioned infectious diseases in the collected news. It also provides news articles and detailed information related to the infectious disease searched for by users. In addition, it displays the number of news articles related to the diseases per district on the map. A higher number of news articles indicates a larger outbreak of infectious disease.
• PEACOCK generates and provides the infection risk level of the searched infectious disease per district on a map by combining two types of infectious disease information: the number of online news articles and the number of portal search queries. In addition, it provides graphs that compare infectious disease outbreak statistics and web data statistics in terms of number, fraction, and difference. It also provides a graph that shows the similarity of disease outbreak statistics to web data statistics. Finally, it presents a graph that shows the match between the systems infection risk values with actual disease outbreak statistics. Consequently, users can visually identify area susceptible to the disease of interest up to one month in advance. They also figure out the accuracy of our system.

To the authors’ knowledge, only a few related systems are in operation [17], [19], [21], [24]. PEACOCK is fully developed and has been in operation since May 2019. It is available online at http://www.epidemic.co.kr/map. Moreover, all figures presented in this work are generated automatically through the system.

## II. Related Works

Existing infectious disease outbreak systems provide information based on various data sources [9]–[12]. Some systems utilize data from government agencies. The Epidemic Simulation System [13] presents infectious disease outbreak information in terms of population and location. It takes population data from the US Census and provides infectious disease outbreak and spread information according to population distribution. Google Dengue Trend [14] shows dengue fever outbreak information for Mexico. It collects data for dengue analysis from the Mexican Health Office. The system collects the Mexican population data through the National Statistics Office and weather data, such as temperature, from the Mexican Secretariat of the Environment and Natural Resources, and it shows the information in graph and map forms. Another system [15] provides influenza outbreak information for China. The system collects official data reported by China’s Ministry of Health and provides influenza outbreak information by comparing the collected data with data predicted by the system. Another system [16] provides malaria outbreak information for Thailand based on the data from the official website of the WHO from 2005 to 2009. The system shows similarities between the outbreak data and its own predicted data.

Other systems utilize online news articles. The Global Public Health Intelligence Network [17] collects news articles from the web, analyzes them, and displays them on its website. In addition, it reconstructs collected news data, filters them, and delivers the necessary information to users through e-mail. EpiSPIDER [18] extracts infectious disease outbreak information, such as keywords and dates, related to diseases among data collected from online news articles. It displays the data on the map using colors based on the elapsed date. It also shows the trend of each disease for three years in a bar graph form. The Medical Information System [19], [20] retrieves online news articles about the disease and displays them according to the user’s search. Based on the number of articles, the system shows the most-common illnesses for each district in a chart form. The user can receive the search result via SMS or e-mail. HealthMap [21], [22] is one of the systems that provide disease outbreak information on a map. The system collects the disease outbreak information in real time from online news articles and processes them as necessary information; it then uses that information to visualize the risk level on the map. Another system [23] provides news media information using HealthMap. It provides the dengue fever outbreak information of Sri Lanka from 2007 to 2015. The system collects online news articles using keywords of both Sri Lanka and dengue-fever-related disease from the HealthMap database, and the number of times that a certain keyword is mentioned in news articles is shown on hourly and monthly graphs. The Program for Monitoring Emerging Diseases Mail [24]–[26] analyzes and extracts online news articles and provides disease outbreak information via the web or e-mail by dividing the risk levels into colors based on the analysis results.

Some systems collect portal search query data and extract infectious disease outbreak information. GET WELL [27] analyzes query logs of web-based search engines in real-time and provides disease outbreak information to users. It collects search query data from some of Sweden’s leading disease-related websites and Google. Google Flu Trends [28], [29] analyzes Google search query data and predicts flu outbreaks. It also compares those prediction results with actual CDC data. Google Dengue Trends uses Google search query data to provide dengue outbreak information in a similar way to Google Flu Trends. It provides disease outbreak information based on anonymous Google search query logs of Mexico in real time, and it shows the risk level in colors on the map. It also compares the prediction results with actual CDC data. A system [16] that shows the risk-level information for malaria in Thailand uses Google search query data and Google Correlate, an open-source search tool. It collects search query data using malaria-related keywords and extracts time-series data obtained using these search keywords. It generates and provides malaria outbreak information to Thai medical practitioners. A system that provides influenza outbreak information for China [30] collects search query data from Baidu, a representative Chinese search engine. In particular, the system uses Baidu’s keyword tool to extract relevant keywords for flu, and users can visualize the results in graph and table forms. The system reported in another study [31] uses search query data from UpToDate, a specialized database in which practitioners upload medical activity. It uses UpToDate to collect search keywords related to influenza-like illness, displays the search ratio of each keyword in bar graph form, and analyzes the ratio to predict influenza outbreaks.

TABLE 1 presents an in-depth comparison of the related works. Many researchers have developed effective infectious disease outbreak information systems, but many of these systems provide outbreak information for only one or a few infectious diseases, exploit only a few kinds of data sources, provide only text-centric information, have not yet been implemented, or are not in operation. However, our system provides outbreak information for as many diseases as possible, exploits various data sources such as CDC, online news, and web search queries, uses various visualization tools such as maps and figures rather than text, and is fully developed and in operation.

**TABLE 1:** List of Existing Systems

System/study	Country	Disease	Data	Data Source	Interface (tool)	In operation
EpiSimS [13]	USA	Influenza	Official cases	US Census, infrastructure data	Simulation program	X
Google Dengue Trend [14]	Mexico	Dengue fever	Official cases, population data, weather data, search query	Mexican Health Office, National Statistics Office, Mexican Secretariat of the Environment and Natural Resources, Google search query	Web (graph, map)	X
[16]	Thailand	Malaria	Official cases, search query	WHO, Google search query	None (paper)	X
GPHIN [17]	Canada	All	News data	Local newspaper, newsletters	Web, email alert	O
EpiSPIDER [18]	USA	All	Official cases, News data, SNS data	CIA, ProMED mail	Web (graph, map)	X
MedISys [19], [20]	EU	All	News data	Europe Media Monitor	Web (text, graph), email or SMS alert	O
Healthmap [21], [22]	USA	All	Official cases, News data	Google news, ProMED Mail	Web (graph, map), email alert	O
[23]	Sri Lanka	Dengue fever	News data	Healthmap	graph	X
ProMED mail[24]–[26]	USA	All	News data	Web sites of ministries of health, WHO, State and local health departments	Web, email alert	O
GET WELL [27]	Sweden	All	Search query	Sweden’s leading disease-related websites, Google search query	Web	X
Google Flu Trends [28], [29]	United States	Influenza	Official cases, search query	CDC, Google search query	Web (graph, map)	X
[30]	China	Influenza	Official cases, search query	China’s Ministry of Health, Baidu	None (paper)	X
[31]	United States	Influenza	Official cases, search query	CDC, Google Flu Trends, UpToDate	None (paper)	X
PEACOCK (Our proposed System)	South Korea	All	Official cases, News data, search query	KCDC, Naver news API, Naver search trend platform	Web (graph, map)	O

## III. System Architecture

**FIGURE 1.**
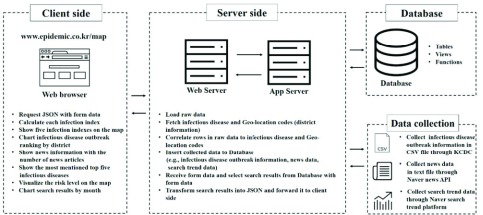
System architecture.

Fig. 1 presents the overall architecture and flow of the proposed system. It was implemented in the Eclipse Jee Oxygen integrated development environment [32] using Java [33] and a web-based client-side interface using HTML5 [34] and CSS [35]. Web pages were dynamically implemented in JavaScript [36] and jQuery [37]. Asynchronous JavaScript and XML (AJAX) [38] data transmission using jQuery was employed. The server-side of the system consists of a web server and an application server. Jetty was used as the web server and the Spring framework [39] as the application server. Finally, PostgreSQL [40] was used as the database management system. The data collection, client side, server side, and database are described below.

### A. Data Collection

The system provides information based on the number of infected people. To provide that information to the user, statistical infectious disease outbreak data provided by KCDC [41] per district are collected in comma-separated values (CSV) [42] file format. The system also provides infectious disease news information and statistics on a map. News articles are crawled using the Naver News API [43] and stored in the form of a text file. Portal search query data are collected using the Naver search trend platform [44].

### B. Client Side

The client side is the user interface. The system provides a form for users to carry out a search by selecting the year, month, district, infectious disease name, and infectious disease outbreak information type. After a user selects items on this search form and submits a request, the request is sent to the web server. First, when the user searches for the number of infected people, the client side calculates the infection indices using input data, the number of infected people, the population of the district, and the number of infected people during the past month. TABLE 2 shows the notation meanings and formulas of infection indices. Once the infection indices are calculated, the client side displays the information on the map. The Naver Map API is used for map implementation. If the user searches the district through the interface, the client-side receives the latitude and longitude data of the district stored in the database from the server, which is displayed in the map. The map is visualized in blue to specify a decreasing monthly infection index, green for no change, and red for an increase. The infectious disease outbreak rankings with the number of infected people are shown by district using a bar chart. The chart is obtained using Chart.js [45], an open-source library that provides various functions for drawing charts. Second, if the user searches for news information, the client side displays the number of news articles by district on the map. It also displays news articles in a table format. In addition, the client-side also shows the five most mentioned infectious diseases in a block form. The client side finally shows the risk level on the map based on the number of infected people, the number of news articles, and the number of search queries. It shows the risk level on the map by combining the aforementioned infection indices, infected-people percentage, news article percentage, and search query percentage. It displays the number of infected people, the number of news articles, and the number of search queries by month through a line chart using Chart.js. It also shows the relative percentages of these parameters so that the user can more easily understand their similarities. The client then calculates the risk level using the relative percentages as follows. TABLE 2 provides the notation meanings and formulas for risk-level calculations. The client side divides the risk into three levels based on the value. If the risk is more than 70% (Risk >70%), red color is used. If it exceeds 50% (Risk >50%), yellow is used. Red represents danger, yellow represents attention, and blue means normal.

**TABLE 2:** Notation Meaning and Formula

Notation	Meaning	Notation	Formula
$N_{Ip}$	Number of infected people that user searched	$I_{u}$	$\frac {N_{IP}}{N_{UIp}} \times 100$
$N_{UIp}$	Number of infected people of upper district of the district that user searched		
$N_{PIp}$	Number of infected people of previous month of the month that user searched	$I_{M}$	$\pm \left({\frac {N_{Ip}}{N_{pIp}} \times 100}\right)$
$N_{Dp}$	Population of the searched district		
$N_{NIp}$	Number of infected people of the nation	$I_{D}$	$\left({\frac {N_{IP}}{N_{Dp}} \times 100000}\right)\times 100$
$I_{u}$	Upper infection index		
$I_{M}$	Monthly infection index	$I_{N}$	$\frac {N_{Ip}}{N_{NIp}} \times 100$
$I_{D}$	District infection index		
$I_{N}$	Nationwide infection index		
$P_{Ip}$	Percent of infected people	$P_{Ip}$	$\frac {N_{Ip}}{N_{IpMax}} \times 100$
$P_{NC}$	Percent of News count		
$P_{SQ}$	Percent of Search query	$P_{NC}$	$\frac {N_{Ip}}{N_{NCMax}} \times 100$
$N_{IpMax}$	Maximum number of monthly infected people during the year		
$N_{NCMax}$	Maximum number of monthly news count during the year	$P_{SQ}$	$\frac {N_{Ip}}{N_{SQMax}} \times 100^{}$
$N_{SQMax}$	Maximum number of monthly search query during the year		

### C. Server Side

The server side extracts the data necessary to calculate the infection index from the CSV statistics file collected from KCDC. The server uses the Java API, POI-HSSF [46], to retrieve the month, infectious disease, district, and number of infected people from the CSV file. It also stores the number of infected people data in connection with infectious diseases and district codes stored in the database. The server uses the Naver News API to fetch and produce news information. It retrieves news data every 30 min by specifying the news keyword as an infectious disease name, saves the articles as a text file with date, and extracts and stores the text in the database. According to the user input, a query fetches the number of news articles mentioned and the news data from the database. It then returns the results received from the database to the client through JSON [47]. When the user retrieves the risk-level information, the server retrieves these three types of information and delivers them to the client. The number of infected persons and the number of news referrals are taken from each table in the database, and search trend data are obtained by entering keywords into the Naver search trend platform. Certain infectious diseases have several names or aliases, and the system includes such names as keywords too.

### D. Database

**FIGURE 2.**
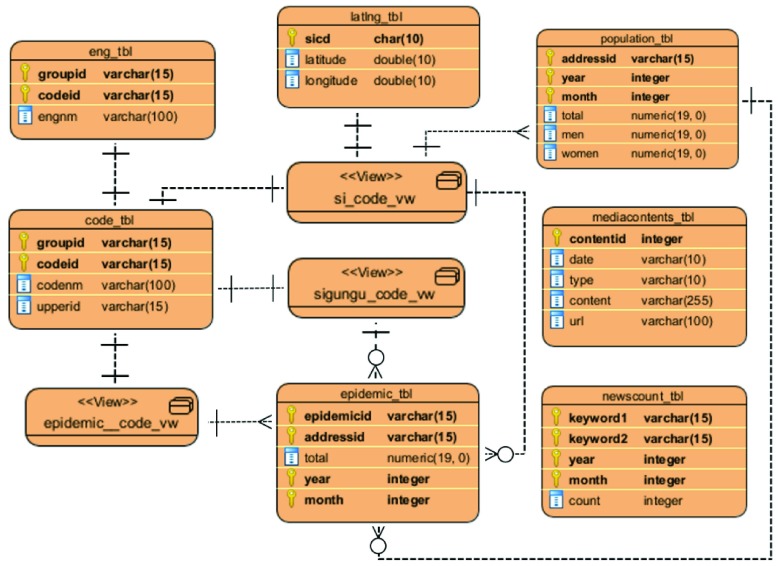
Entity relationship diagram.

The system stores the collected data in each table of the database. TABLE 3 shows a detailed description of each table and view [48] of our database. Seven tables and three views were created. Views were created to include only the desired data to implement fast retrieval. Fig. 2 is the entity relationship diagram (ERD) [49] that shows the relationship to each table in the database.

**TABLE 3:** Detailed Description of the Tables

Table	Detail
code_tbl	Groupcode, unique code, name and upper group code of si, sigungu and infectious disease
eng_tbl	Groupcode, unique code and english name of si, sigungu and infectious disease
epidemic_tbl	Number of infected persons per each district and each infectious disease
latlng_tbl	Latitude and longitude of each district
mediacontents_tbl	Infectious disease news information by date (date, content, news url)
newscount_tbl	Date and number of times mentioned in the news per each keyword
population_tbl	Current population of each district by date
si_code_vw	Si-code, unit of Si (district information)
sigungu_code_vw	Sigungu-code, unit of Sigungu (district information)
epidemic_code_vw	Infectious disease-code, infectious disease name

## IV. Results

The system provides the user with three types of infectious disease outbreak information, namely the number of infected people, news information, and risk-level information. The system provides five search options: year, month, district, infectious disease, and type of information. The system provides information in two languages: Korean and English.

### A. Information Based on the Number of Infected People

**FIGURE 3.**
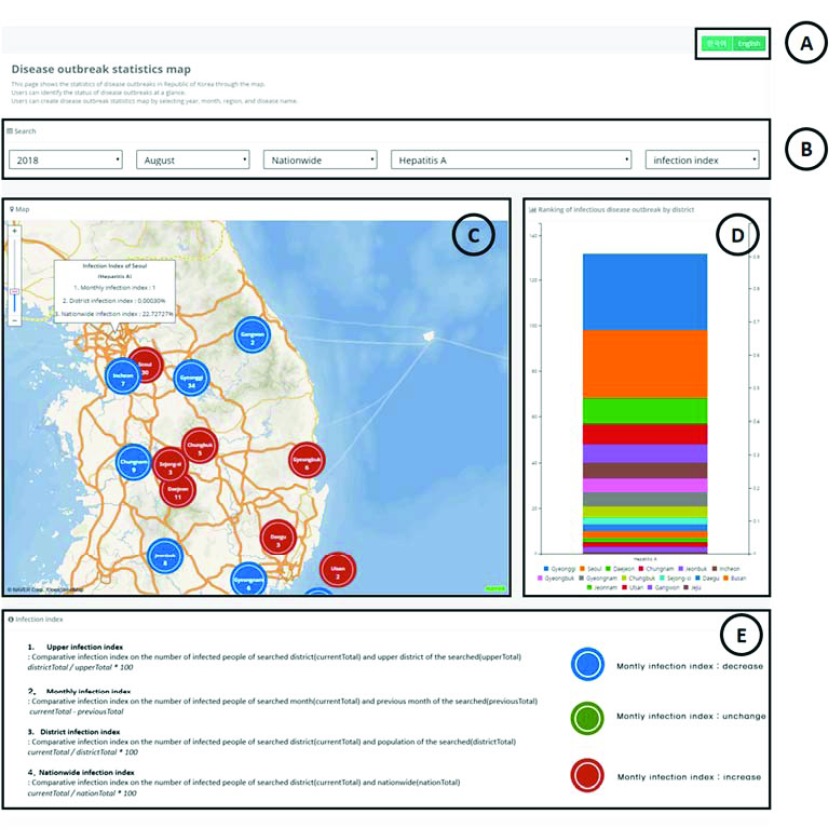
User interface of information based on the number of infected people.

Fig. 3 shows the user interface for information on the number of infected people. Fig. 3A shows the language settings, and Fig. 3B shows the search form. Fig. 3C is the map displaying infectious disease outbreak statistics. Through this map, the user can quickly view whether the number of infected people has increased or decreased compared with the previous month. The user can see the number of infected people in each district. When the user hovers the cursor over any district, the system shows four infection indices: the upper infection index, monthly infection index, district infection index, and nationwide infection index. Fig. 3D is a chart showing the ranking of the districts in the order of number of infected people. Fig. 3E presents the description of each infection index.

### B. Information Based on Online News

**FIGURE 4.**
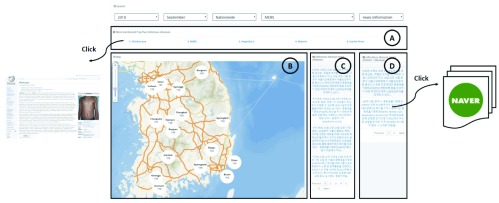
User interface of information based on online news.

Fig. 4 shows the user interface of the news-based information. Fig. 4A shows the top-five infectious diseases mentioned in the news during a particular month. The user can see detailed information, such as definitions, symptoms, and prevention methods, of the infectious diseases. Fig. 4B shows the map with the number of news articles on the searched infectious disease. This information enables the user to predict the likelihood of an outbreak of an infectious disease. Fig. 4C shows highlights of the news about the searched infectious diseases. The user can see the news in the order of the most recent date during a particular month. In addition, the user can also check news on the infectious disease news per district, as seen in Fig. 4D. The user can quickly view each news item briefly, and, when it is clicked, the user can see the entire article.

### C. Risk Level Information

**FIGURE 5.**
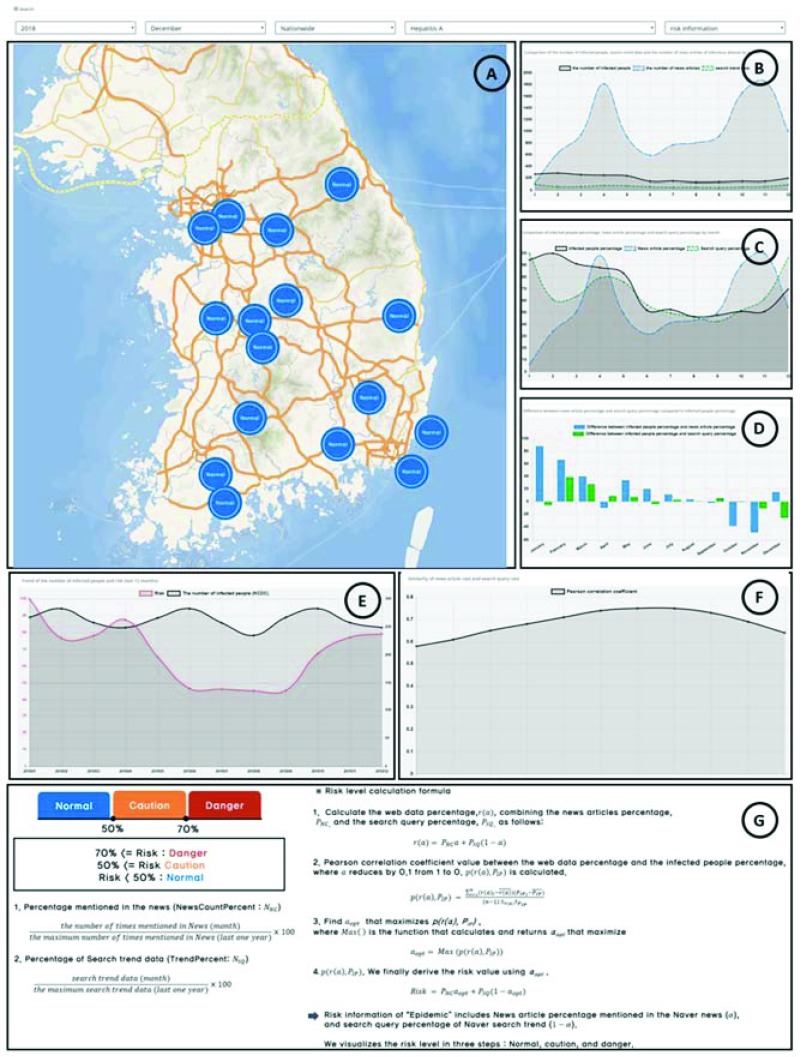
User interface of risk level information.

Fig. 5 shows the user interface of the risk-level information. In Fig. 5A, when users input year, month, district, and infectious disease in the search form, the system divides the risk into three levels, namely normal, attention and risk, and it displays them as blue, orange, and red, respectively. Fig. 5B compares the actual numbers of infected people, news articles, and search queries as a function of month. The actual number is important, but it is not appropriate to compare the similarities of different data types because the difference in the size of the number may be too large. Fig. 5C shows the comparison of the relative percentages of the three aforementioned data. The relative percentage is defined as the numbers of the selected month over the maximum number of the year, which helps to understand the similarities of different data types. Fig. 5D illustrates the difference between news article percentage and infected-people percentage, and the difference between search query percentage and infected-people percentage. In Fig. 5B to D, the graph can be removed or redrawn by clicking on each legend. Fig. 5E evaluates the accuracy of the risk value by comparing the risk value with the infected people percentage. Fig. 5F presents the Pearson correlation coefficient between the infected people percentage and the web data percentage calculated by equations (2) and (3).

Fig. 5G provides a detailed description of the risk level.

## V. Analysis

In this section, we analyze our proposed system and evaluate the similarities between the actual infected people data and the web data (i.e., news article, search query) and the accuracy of the risk-level equation proposed in the system.

**FIGURE 6.**
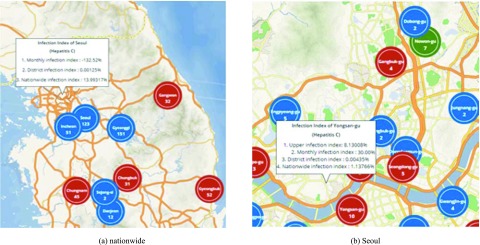
Information based on the number of infected people.

Figs. 6A and B show the infection index information on the map. Fig. 6A shows the nationwide infection index of hepatitis C in August 2018. In the case of Seoul, the upper infection index and the nationwide infection index are the same, because the upper district of Seoul is representative of the entire nation. Therefore, the system provides three infection indices, monthly, district and nationwide, when the keyword of the nationwide is selected. The nationwide infection index is 12.99317, which is higher than the national average. The monthly infection index is −132.52, and the district infection index is 0.00125. Fig. 6A shows the hepatitis C infection index in Seoul in August 2018. Most of Seoul’s subdistricts also have reduced number of infected people. In addition, the infection index is low in Seoul. However, the upper infection index is 8.13008 in Yongsan district. Users can pinpoint not only the number of infected people by district, but also their relative meaning in terms of the district, time, and population.

**FIGURE 7.**
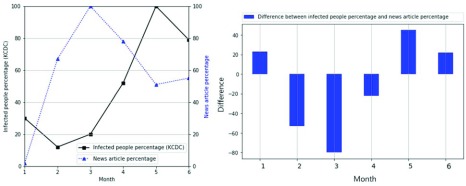
Similarity of infected people percentage and news article percentage of Scrub typhus as a function of month.

**FIGURE 8.**
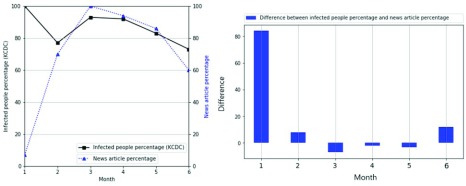
Similarity of infected people percentage and news article percentage of hepatitis C as a function of month.

Figs. 7 and 8 compare the infected people percentage with the news article percentage. The x-axis represents the month, and the y-axis represents the percentage in the line graph. The black bold square line indicates the infection percentage, while the blue triangular dotted line indicates the news article percentage. The blue bar is the difference between the news article percentage and the infected people percentage in the bar graph. Fig. 7 compares the infected people percentage with the news article percentage of Scrub typhus from January to June 2018, where the trend of the news article percentage is different from that of the infected people percentage. This is because the news contains intentional information, such as advertisements [50]–[52]. The bar figure has many large bars, which indicates a large difference. Fig. 8 compares the infection percentage with the news article percentage of hepatitis C from January to June 2018. Unlike the trend of Scrub typhus in Fig. 7, except for January, hepatitis C shows a similar trend. The bar figure has few large bars, which indicates similarity. Thus, the news query percentage is sometimes similar to the infection percentage, so it can be used to estimate the infected people percentage.

**FIGURE 9.**
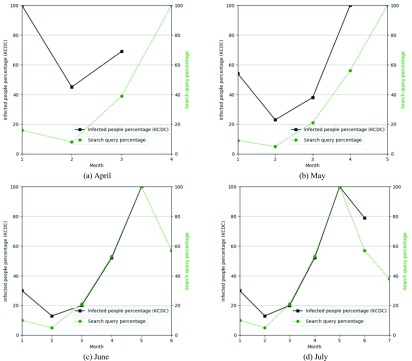
Similarity of infected people percentage and search query percentage of scrub typhus by month: (a) April, (b) May, (c) June and (d) July.

Fig. 9 compares the infected people percentage with the search query percentage of Scrub typhus from January to July 2018. Because the KCDC data are uploaded once a month, if the user searches for July, the infected people percentage is displayed until June. However, because the search query percentage is provided in real time, it includes data for July. Fig. 9 shows that the infected people percentages are very similar to the search query percentages. Users can predict that the infected people percentage decreases based on the search query percentage in July. As a result, the search query percentage can be used to predict the infected people percentage.

To show the similarity in the percentages clearly, we provide a correlation graph between the infected people percentage and the web data percentage for each infectious disease. We use the Pearson correlation model to derive the correlation. The Pearson correlation analysis model is a method of expressing the similarity between two changing data sets, $a$ and $b$, as numerical values $p$, as follows:

\begin{equation*} p(a,b)=\frac {\sum \nolimits _{i=1}^{n} {(a_{i}-\overline a)(b_{i}-\overline b)}}{\left ({n-1 }\right) S_{a}S_{b}}\tag{1}\end{equation*}

where n is the length of $a$ and $b$, $\overline a $ and $\overline b $ are sample averages for each data set $a$ and $b$, and $S_{a}$ and $S_{b}$ are the standard deviations for the two data sets. The resulting $p$ values range between −1 and 1, and the closer the value is to 1, the higher the positive correlation.

**FIGURE 10.**
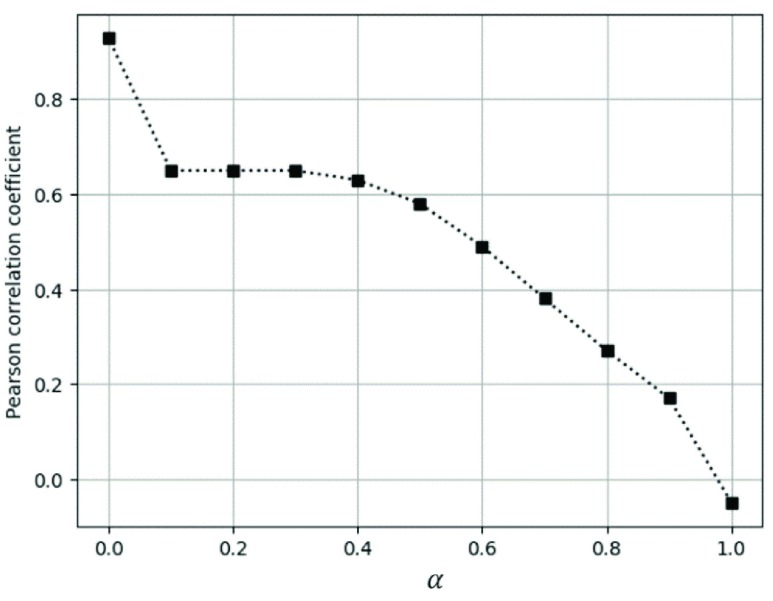
Pearson correlation coefficient between the infected people percentage and the web data percentage calculated by the equation (2) and (3) of scarlet fever according to $\boldsymbol {\alpha }$.

Fig. 10 shows $p(r\left ({a }\right), P_{IP})$, the Pearson correlation coefficient values between the infected people percentage and web data percentage for the Scarlet fever according to $\alpha $ values. We calculate the web data percentage, $r(a)$, by combining the news articles percentage, $P_{NC}$, and the search query percentage, $P_{SQ}$, as follows:

\begin{equation*} r(a)= P_{NC}a+P_{SQ}(1-a)\tag{2}\end{equation*}

As the value of $\alpha $ increases, the proportion of the news percentage increases and the proportion of the search query percentage decreases. By contrast, as the value of $\alpha $ decreases, the proportion of the news percentage decreases, and the proportion of the search query percentage increases. Fig. 10 shows that the scarlet fever outbreak is inversely proportional to the news percentage, while conversely, we can confirm that scarlet fever outbreak correlates well with the search query percentage.

**FIGURE 11.**
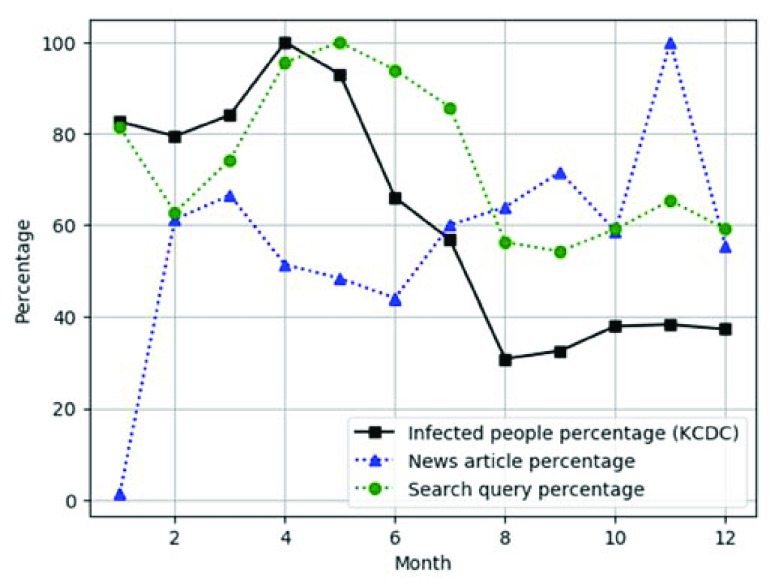
Similarity of infected people percentage, news article percentage and search query percentage of scarlet fever as a function of month (2018).

Fig. 11 presents the infected people percentage with the news article percentage and the search query percentage for scarlet fever. The x-axis represents the month, and the y-axis the percentage. The black bold square line indicates the infected people percentage, the blue triangular dotted line indicates the news article percentage, and the green circular dotted line indicates the search query percentage. As can be seen in Fig. 10 and Fig. 11, the incidence of scarlet fever differs significantly from the news article percentage and shows a similar trend to the search query percentage. Hence, we can identify the outbreak of each disease through web data by checking the similarity between the infected people percentage of each infectious disease and the web data (i.e., news and search query) percentages through given correlation analysis graph before the comparatively time-consuming KCDC data are collected.

Finally, to predict the outbreak of each infectious disease, we calculate the risk values and visualize them on the map by optimally combining the web data percentages, such as the news article percentage and the search query percentage. First, we derive the risk by calculating $p(r\left ({a }\right), P_{IP})$, i.e., the Pearson correlation coefficient value between the web data percentage and the infected people percentage, where $a$ reduces by 0.1 from 1 to 0. $p(r\left ({a }\right), P_{IP}) $ is calculated as follows:

\begin{equation*} p\left ({r(a), P_{I P}}\right)=\frac {\sum _{i=1}^{n}\left ({r(a)_{i}-\overline {r(a)}}\right)\left ({P_{I P i}-\overline {P_{I P}}}\right)}{(n-1) S_{r(a)} S_{P_{I P}}}\tag{3}\end{equation*}

Equation (3) helps to obtain the correlation coefficient between the actual number of outbreaks and the web data frequency according to the change in $a$. We find $a_{opt}$ that maximizes $p(r\left ({a }\right),P_{IP}) $ as follows.

\begin{equation*} a_{opt}= Max (p(r\left ({a }\right), P_{IP}))\tag{4}\end{equation*}

where $Max\left ({}\right)$ is the function that calculates and returns $\alpha _{opt}$ that maximizes $p(r\left ({a }\right), P_{IP})$. We finally derive the risk value using $\alpha _{opt}$ as follows.

\begin{equation*} Risk = P_{NC}a_{opt}+P_{SQ}(1-a_{opt})\tag{5}\end{equation*}

**FIGURE 12.**
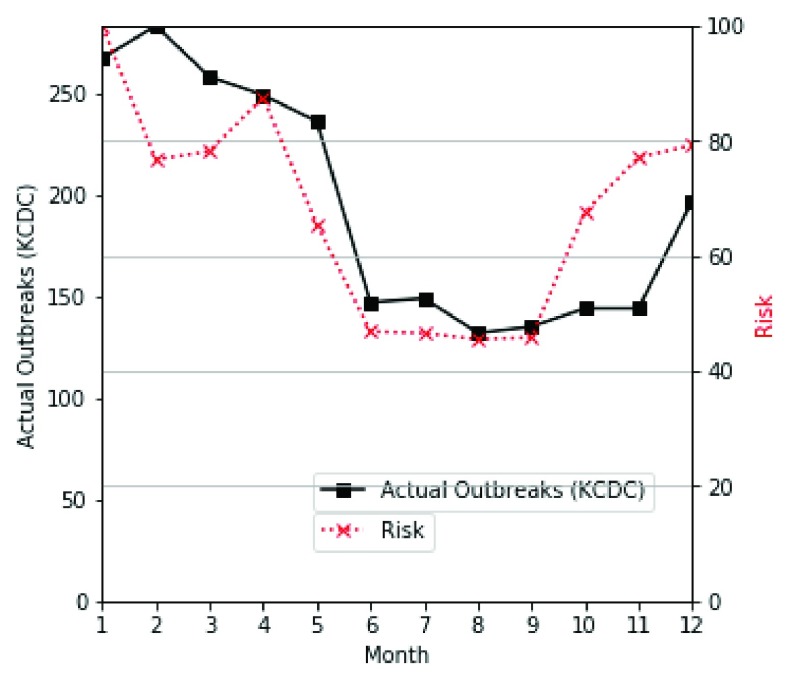
Similarity of actual outbreaks and risk of hepatitis A as a function of month (2018).

Our system shows the similarity of our proposed risk value to the number of infected people provided by the KCDC. Fig. 12 shows the actual outbreak frequency and risk value of hepatitis A in 2018. The black bold square line is the actual number of occurrence, and the blue dotted line is the risk level. We can confirm that the risk value is fairly similar to the actual number of outbreaks of the infectious disease from KCDC.

**FIGURE 13.**
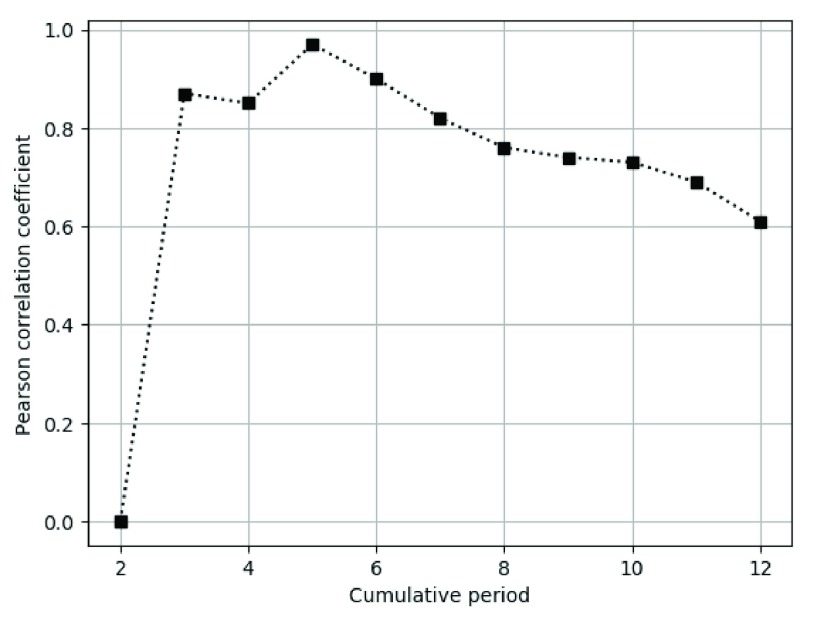
Pearson correlation coefficient between infected people percentage and risk of hepatitis A by cumulative period.

For accurate similarity analysis, we used the Pearson correlation coefficient and divide this trend by monthly cumulative period (1 month, 2 months, etc., up to 12 months). Fig. 13 shows a low degree of similarity initially, but as the period increases, the similarity improves.

Table 4 shows the value of the Pearson correlation coefficient between actual outbreaks and risk values for top twenty common infectious diseases in South Korea during 2018. Most of them show high correlation coefficient, which shows that our proposed risk equation is fairly accurate.

**TABLE 4:** Pearson Correlation Coefficient Between Actual Outbreaks and Risk for Common 20 Infectious Disease

Infectious disease	Pearson correlation	Infectious disease	Pearson correlation
Chicken pox	0.61	Streptoccoccus pneumonia	−0.03
Epidemic parotitis	0.68	SFRS	0.56
Scarlet fever	0.78	Malaria	0.77
Measles	0.84	Acute hepatitis B	0.01
Hepatitis C	0.11	Typhoid fever	0.43
Scrub typhus	0.19	Legionellosis	0.09
Primary Syphilis	−0.4	Brucellosis	0.21
Pertussis	0.77	Dengue fever	0.53
Q fever	0.46	Lyme disease	0.64
Hepatitis A	0.64	Secondary syphilis	−0.16

## VI. Conclusion

Humans continue to suffer from the ongoing outbreak of infectious diseases. The recent spread of mobile devices has increased the importance of infectious-disease outbreak information systems that aggregate data related to these diseases and provide outbreak information to users accurately and quickly. In this work, a map-based multitype infectious disease outbreak information system was presented that provides information based on the number of infected people, information based on online news, and risk-level information by combining the number of infected people, news items, and search queries. The system depicts the information using maps and various figures, and users can pinpoint the information easily. The system presents various and necessary types of disease information separately, in combination and in comparison. The system will help people monitor and prevent infectious diseases by providing them with necessary infectious disease outbreak information accurately, quickly, and visually through a user-friendly interface. The system is currently available on the web at http://www.epidemic.co.kr/map.

## VII. Future Research Agenda

We have three future ongoing research works. First, we analyze the similarity among actual infectious disease outbreak statistics and various relevant web data statistics. Most related works have analyzed web data for single or few diseases. We analyze web data for as many diseases as possible and try to reveal their patterns of similarities. Second, we find efficient keywords for web data collection using artificial intelligence technologies. Our system collects news and search queries by simply using names for specific diseases. We can find words related to specific diseases using Word2Vec techniques [53], [54]; as a result, we can use them as keywords for the data collection to gather more relevant web data. Third, our final goal is to provide accurate prediction information for infectious disease outbreaks. We develop general prediction models that can be applied to as many diseases as possible rather than specific diseases. Because the characteristics of the diseases are different from each other, it is difficult to create a general model. We classify the infectious diseases and develop models for each disease group. We then apply the models to our system and provide the user with accurate prediction information of infectious disease outbreaks. We believe that our system will provide not only actual infectious disease outbreak information and various related web data statistics but also predictions of infectious disease outbreak in the near future.
